# Astroglial networks control visual responses of superior collicular neurons and sensory-motor behavior

**DOI:** 10.1016/j.celrep.2024.114504

**Published:** 2024-07-13

**Authors:** Josien Visser, Giampaolo Milior, Rachel Breton, Julien Moulard, Maina Garnero, Pascal Ezan, Jérôme Ribot, Nathalie Rouach

**Affiliations:** 1 Neuroglial Interactions in Cerebral Physiology and Pathologies, Center for Interdisciplinary Research in Biology, Collège de France, CNRS, INSERM, Labex Memolife, Université PSL, Paris, France; 2 Doctoral School No. 158, Sorbonne Université, Paris, France

**Keywords:** superior colliculus, visual maps, astrocytes, synaptic circuits, gap junctions, astroglial networks, sensory-motor behavior

## Abstract

Astroglial networks closely interact with neuronal populations, but their functional contribution to neuronal representation of sensory information remains unexplored. The superior colliculus (SC) integrates multi-sensory information by generating distinct spatial patterns of neuronal functional responses to specific sensory stimulation. Here, we report that astrocytes from the mouse SC form extensive networks in the retinorecipient layer compared to visual cortex. This strong astroglial connectivity relies on high expression of gap-junction proteins. Genetic disruption of this connectivity functionally impairs SC retinotopic and orientation preference responses. These alterations are region specific, absent in primary visual cortex, and associated at the circuit level with a specific impairment of collicular neurons synaptic transmission. This has implications for SC-related visually induced innate behavior, as disrupting astroglial networks impairs light-evoked temporary arrest. Our results indicate that astroglial networks shape synaptic circuit activity underlying SC functional visual responses and play a crucial role in integrating visual cues to drive sensory-motor behavior.

## Highlights

• Astrocytes in superior colliculus (SC) form extensive networks via gap junctions
• This connectivity shapes spatial patterns of neuronal visual responses in the SC
• This astroglial regulation is specific to SC, as it is not found in visual cortex
• Astroglial networks promote SC-dependent visually induced innate behavior

## Graphical abstract

**Figure:**
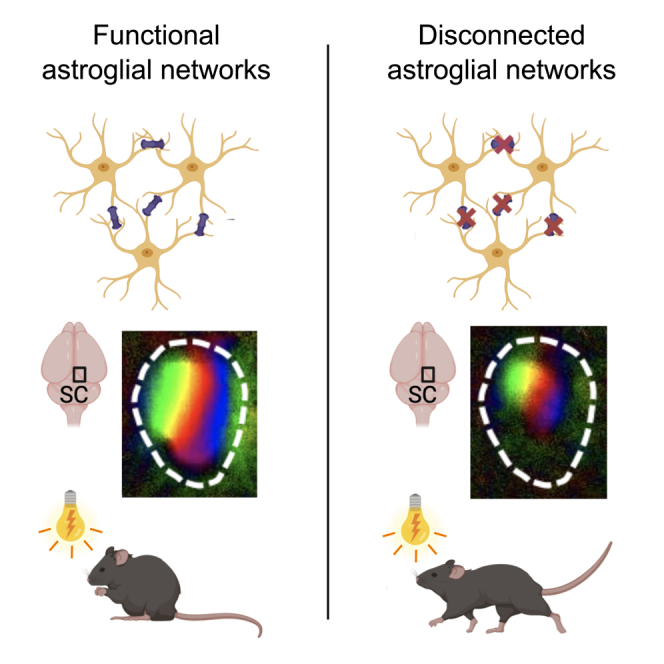

## Teaser

The superior colliculus (SC) is an evolutionary conserved sensory-motor region integrating multi-sensory information into specific spatial patterns of neuronal responses. Visser et al. found that the extensive astroglial collicular networks shape neuronal visual responses of retinotopy and orientation. Astroglial connectivity and regulation are SC specific and promote SC-dependent visually induced behavior.

## Introduction

Astrocytes and their network organization are an integrated component of brain circuits and can influence neuronal network activity.^1^^,^^2^ As a result, there is an emerging view that brain function and cognitive processes arise from dynamic neuroglial interactions.^3^ Astrocytes have also been reported to modulate sensory processing, as sensory-induced astroglial calcium responses have been shown in different areas, including the primary visual (V1) and somatosensory cortices,^4^^,^^5^ and optogenetic activation of astrocytes regulates *in vivo* response selectivity of V1 neurons^6^ and the sensory-evoked gamma activity in the somatosensory cortex.^5^

Interestingly, astrocytes regulate synaptic connectivity between the retina and the superior colliculus (SC),^7^ a structure in the mammalian midbrain that is a central part of the visual system, via secreted proteins such as SPARC and Hevin.^8^ The SC is involved in complex behaviors by transforming multisensory information into motor outputs.^9^^,^^10^ However, little is known about the role of astrocytes in the function of the SC.

The mouse SC has recently emerged as a particularly relevant model for studying neuronal network responses in visual processing^11^ and their organization into functional maps. Indeed, when stimulated with oriented bars, neurons in the mouse SC are selective to orientation.^12^ Moreover, neighboring neurons share similar orientation preference, forming a functional map parallel to the surface.^13^^,^^14^ This is precisely as in the V1 of other mammals such as cats,^15^ but not as the V1 of rodents, where neurons sharing the same orientation preference appear to be randomly distributed across the cortical surface.^16^ It is noteworthy that functional maps do not only rely on the static anatomical neuronal connections, but also on the dynamic responsiveness of these connections to visual stimuli. Although astrocytes can respond to visual sensory stimuli and, in turn, modulate neuronal sensory-evoked responses, whether they contribute to functional visual maps is unknown. Here, we show that the extensive connectivity of astrocytes in the SC control functional visual maps in an activity-dependent manner and sensory-motor behavior.

## Results

### Extensive and non-compartmentalized astroglial networks in the visual layers of the SC

**Figure 1:**
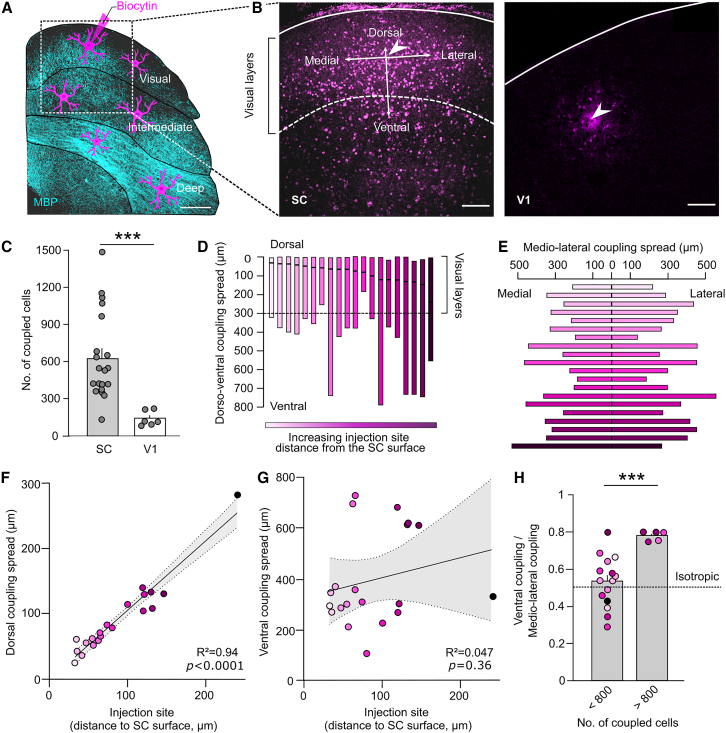
Astrocytes form extensive and non-compartmentalized networks in the visual layers of the SC (A and B) Schematic representation of the experimental approach (A), where GFP-labeled astrocytes from GFAP-EGFP mice were patched with a pipette filled with biocytin to visualize the astroglial network in the visual layers of the SC (B, left) or in V1 (B, right). The SC layers were visualized with myelin basic protein (MBP) stain. The white lines indicate the surface of the SC and V1, respectively, whereas the dotted line shows the borders of the visual layers in the SC. The white arrowheads indicate the injection site. The coupling (median distance of coupled cells from the injection site) for all 4 directions are represented with white arrows. Scale bars, 250 μm (A) and 100 μm (B). (C) Quantification of the size of the astroglial networks in the SC (*n* = 20) and in V1 (*n* = 6; *p* < 0.0001, *U* = 2, Mann-Whitney test). (D) Dorsoventral coupling in astrocytic networks. Injection site (black line) ± maximal extent (color) of coupled cells into dorsal and ventral directions. The astroglial dorsoventral coupling is plotted against increasing injection site distance from the SC surface. The dotted line represents the visual layers border. (E) Medio-lateral coupling of astroglial networks. Injection site ± maximal extent (color) of coupled cells into medial and lateral directions. The astroglial medio-lateral coupling is plotted against increasing injection site distance from the SC surface. (F and G) Astroglial network coupling in the dorsal (F) and ventral (G) directions with respect to the injection site distance to the SC surface. Linear regression with 95% confidence intervals (dorsal: *R*^*2*^ = 0.9352, F(1, 18) = 259.7, *p* < 0.0001, *n* = 20; ventral: *R*^*2*^ = 0.047, F(1, 18) = 0.8882, *p* = 0.3584, *n* = 20). (H) Ratio between the ventral coupling and the medio-lateral coupling for small (<800 coupled cells; *n* = 15) and large (>800; *n* = 5) astrocytic networks (*p* = 0.0009, *U* = 3, Mann-Whitney test). Data are shown as mean ± SEM.

We investigated the spatial properties of astroglial networks in the visual layers of the SC. To do so, we performed patch-clamp recordings of GFP^+^ astrocytes from GFAP-EGFP mice with a pipette filled with biocytin, a gap junction channel permeable tracer, and observed diffusion into neighboring cells (Figure 1A). Astrocytes in this structure displayed characteristic electrophysiological properties,^17^ including passive currents, low membrane resting potential and resistance, and typical capacitance (Figure S1). However, we observed unusual large astroglial networks in the retinorecipient SC. For comparison, we performed similar recordings in another visual area, V1, and found that the SC had about 4 times more coupled cells (629.8 ± 78.1, *n* = 20) than V1 (141.5 ± 60.81, *n* = 6; *p* < 0.0001, *U* = 2, Mann-Whitney test; Figures 1B and 1C).

We then examined whether these networks were compartmentalized within the retinorecipient layer of the SC by analyzing the dorsoventral astrocytic coupling (Figure 1D). We found that the spread into the dorsal direction was typically smaller than the spread into the ventral direction (*p* < 0.0001, Wilcoxon signed rank test, *n* = 20). This contrasts with the medio-lateral coupling, where no difference could be found between the two directions (Figure 1E, *p* = 0.84, Wilcoxon signed rank test, *n* = 20). These results suggest that astroglial networks in the visual layers of the SC are anatomically restricted by the SC surface dorsally, but not by the border between superficial and intermediate layers. To further test the latter point, we calculated the correlation between the injection site distance and the coupling spread into dorsal (Figure 1F) and ventral (Figure 1G) directions. In case of compartmentalization, the border between superficial and intermediate layers should also create a restriction for the astroglial networks’ coupling. We observed that patching a cell more distant from the SC border correlated with the dorsal coupling, as the astroglial networks had more space to extend toward the SC border (Figure 1F; *R*^*2*^ = 0.9352, F(1, 18) = 259.7, *p* < 0.0001, *n* = 20, linear regression). Conversely, we found no correlation between ventral coupling and the location of the injection site (Figure 1G; *R*^*2*^ = 0.047, F(1, 18) = 0.8882, *p* = 0.3584, *n* = 20). Furthermore, the extent of the astroglial network toward the dorsal and the lateral directions was isotropic for networks with less than 800 coupled cells (0.533 ± 0.137, *p* = 0.367, 1-sample t test against 0.5), and was biased toward the dorsal direction for networks with more than 800 coupled cells (*n* = 5; *p* < 0.0009, *U* = 3, Mann-Whitney test; Figure 1H).

Together, these data indicate that the extensive astroglial networks in the visual layers of the SC are not compartmentalized to the visual layers and extend into deeper layers.

### The strong connectivity of SC astroglial networks is mediated by high levels of gap junction channels

**Figure 2:**
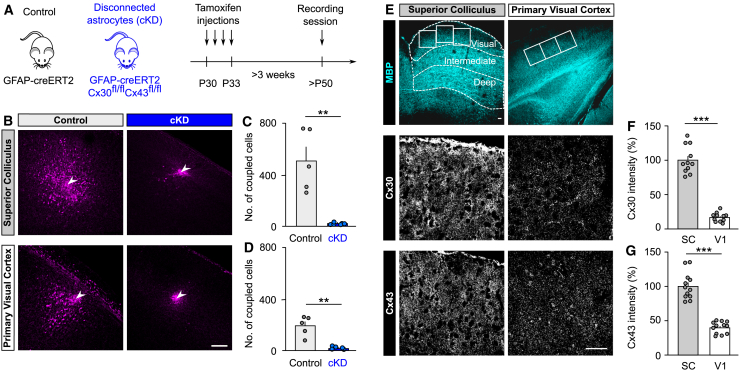
The extensive astroglial network in the SC is mediated by high levels of Cx30 and Cx43 expression (A) Mice model used for control (GFAP-creERT2 + tamoxifen) and disconnected astrocytes (GFAP-creERT2 Cx30^fl/fl^Cx43^fl/fl^ [cKD] + tamoxifen) conditions. Tamoxifen was injected in control and cKD mice during 4 consecutive days, and experiments were performed at least 3 weeks after the last injection. (B) Representative examples of astroglial networks in the visual layers of the SC (top) and in V1 (bottom) for control (left) and cKD (right) mice. (C and D) Quantification of the size of the astroglial networks in the SC (C) and in V1 (D) between control and cKD mice. SC: control, *n* = 5; cKD, *n* = 6; *p* < 0.0043, *U* = 0, Mann-Whitney test. V1: control, *n* = 5; cKD, *n* = 5; *p* = 0.0079, *U* = 0, Mann-Whitney test. (E) Representative images of brain tissues immunostained for MBP (top row, scale bar, 300 μm), Cx30 and Cx43 (center and bottom rows, scale bar, 50 μm) in the SC (left column) and in V1 (right column). Two to three fields of view were imaged per slice (white squares enlarged in center and bottom rows) from 4 mice to quantify Cx30 and Cx43 expression levels. (F and G) Quantification of Cx30 (F) and Cx43 (G) levels in the visual layers of the SC and in V1. Unpaired t test (Cx30 [t = 13.04, *n* = 11 for SC and V1, ^∗∗∗^*p* < 0.0001]; Cx43 [t = 9.768, *n* = 12 for SC and V1, ^∗∗∗^*p* < 0.0001]). Data are shown as mean ± SEM.

We then tested whether the extensive astroglial connectivity found in the SC is mediated by gap junction channels composed of the astrocyte subunit proteins connexins 30 (Cx30) and 43 (Cx43).^18^ To do so, we generated an astroglial conditional and inducible Cx30/Cx43 knockdown mice (GFAP-creERT2 Cx30^fl/fl^/Cx43^fl/fl^, cKD) (Figure 2A), in which the expression of gap junction subunit proteins is reduced by ∼75% for Cx30 and 90% for Cx43 in astrocytes from adult SC (Figure S2). We found that the functional astroglial connectivity in the SC and in V1 was inhibited in cKD mice compared to control mice (GFAP-creERT2, *n* = 18) (Figures 2B–2D; SC: control, *n* = 5; cKD, *n* = 6; *p* < 0.0043, *U* = 0, Mann-Whitney test; V1: control, *n* = 5; cKD, *n* = 5; *p* = 0.0079, *U* = 0, Mann-Whitney test), as assessed by quantifying the size of astroglial networks after biocytin injection (Figures 2B–2D). These data indicate that the large astroglial networks in the visual layers of the SC are mediated by gap junction channels composed of the astroglial Cx43 and Cx30 subunits.

We next investigated whether the strong astroglial connectivity mediated by gap junction channels results from an increased expression in the gap junction subunit proteins specifically in the SC. To this end, we compared the expression patterns of the two astroglial connexins, Cx30 and Cx43, in the visual layers of the SC and in V1. Using immunohistochemistry, we found that both Cx30 and Cx43 were homogenously distributed throughout the visual layers of the SC (Figure 2E). Furthermore, Cx30 and Cx43 were more strongly expressed in the visual layers of the SC (*n* = 11) relative to V1 (*n* = 11) (Figure 2F, Cx30: 16.56% of SC expression, *p* < 0.0001, unpaired t test; Figure 2G, Cx43: 39.68% of SC expression, *p* < 0.0001, unpaired t test). We confirmed these results with western blot, and showed that both Cx30 and Cx43 were expressed at much higher levels in the visual layers of the SC (*n* = 6) relative to V1 (*n* = 5) (Figure S3, Cx30: 29.97% of SC expression, *p* = 0.0004, unpaired t test; Cx43, 27.78% of SC expression, *p* < 0.0001, unpaired t test). This increase in Cx43 and Cx30 expression in the visual layer of the SC reflects an increased expression at the single-cell level, as shown by the quantification of Cx immunostaining per astrocyte (Figure S4, Cx30, t(28) = 12.54, ^∗∗∗^*p* < 0.0001; Cx43, t(28) = 5.142, ^∗∗∗^*p* < 0.0001, unpaired t test). Together, these results indicate the presence of extensive and non-compartmentalized astroglial networks in the visual layers of the SC relying on abundant and homogeneous expression of connexins.

### Astroglial networks are required for functional retinotopic maps in the SC

We next investigated whether the extensive astroglial functional networks contribute to visual maps in the SC. To this end, we used the cKD mice, in which we first tested the retinal functions by recording the electroretinograms. The retinal functions were unaltered in these mice, as indicated by the normal functioning of rods and cones in the retina that were probed with both scotopic and photopic electroretinograms, respectively (Figure S5).

**Figure 3:**
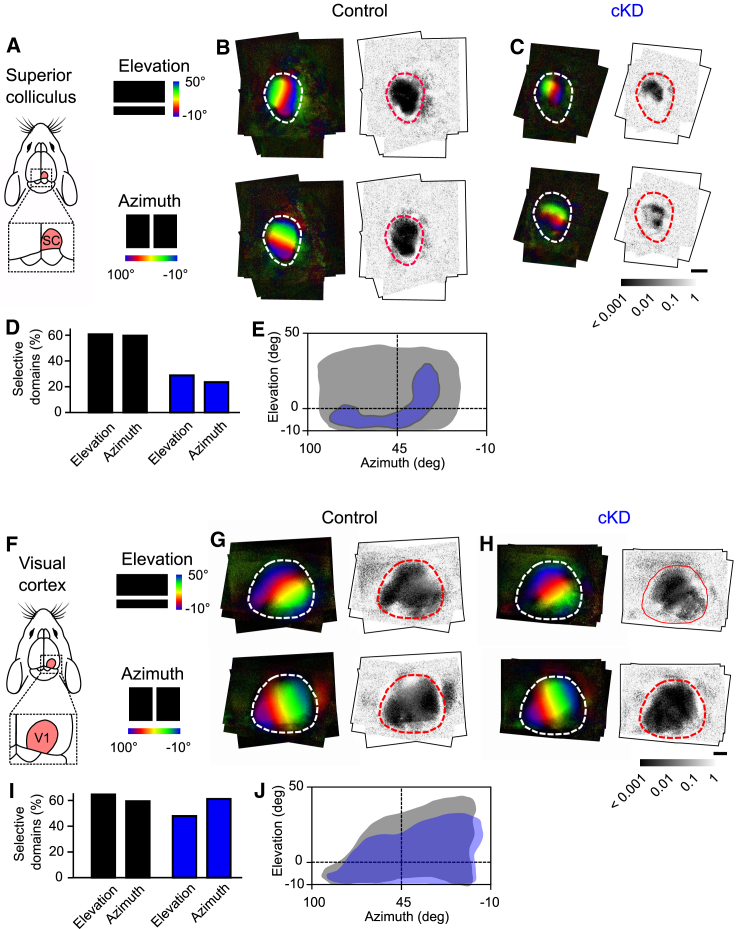
Astroglial networks are required for functional retinotopic maps in the SC (A) Schematic representation of the SC imaged with intrinsic optical imaging. (B and C) Averaged retinotopic maps of elevation (top) and azimuth (bottom) in the SC for (B) control (*n* = 5) and (C) cKD (*n* = 8) mice. Reproducibility in functional organization across animals was tested with the Moore-Rayleigh test (right). Boundaries of the SC is depicted with dotted line. Scale bar, 1 mm. (D) Quantification of collicular domains selective for elevation and azimuth in control (black) and cKD mice (blue). (E) Representation of visual locations selective for both elevation and azimuth in control (gray area) and cKD (blue area) mice. (F–J) Same as (A)–(E) when retinotopic maps are recorded from the visual cortex of control (*n* = 5) and cKD (*n* = 4) mice.

To test for functional modifications in the SC, we assessed its retinotopy, which has been well described in this brain region^19^ and refers to the representation of the visual field within the visual system. We used optical imaging of intrinsic signals and recorded maps of retinotopy for elevation and azimuth (Figures 3A–3C). In control animals (GFAP-CreERT2 mice injected with tamoxifen), we found that the elevation is represented medio-laterally, and the azimuth is mapped along the anterior-posterior axis (Figure 3B, left panels), as reported in wild-type (WT) mice.^19^ These two maps were highly robust over the whole SC (Moore-Rayleigh test; Figure 3B, right panels). In cKD mice (after tamoxifen injection), the retinotopic maps of elevation (top left panel) and azimuth (bottom left panel) appeared incomplete, with only small portions of the visual field that were represented (Figure 3C). Accordingly, selective domains (i.e., with a robust mapping across animals [Figure 3D, Moore-Rayleigh test, *p* < 0.05]) were decreased by ∼50% in cKD mice (*n* = 8) compared to controls (*n* = 5). In particular, only the lower and more frontal parts of the visual field were systematically represented in the SC (Figure 3E). These functional impairments in cKD mice were not due to alterations in map formation during development, as we induced Cx knockdown via tamoxifen injection at postnatal day (P)30–P35, when maps are already established.^20^^,^^21^ We indeed found that prior to tamoxifen injection, cKD mice exhibited similar retinotopic maps compared to control animals (Figure S6).

As astroglial networks are extensive in the SC, we investigated whether they specifically contribute to retinotopic maps in this brain area. We thus assessed retinotopy in V1 and found that impairing astrocytic connectivity in cKD mice had no effect compared to control animals (Figures 3F–3J). Typical retinotopic maps were consistently found in V1 of both cKD and control animals (Figure 3I), and representation of these maps in the visual field shows that retinotopy largely overlapped (Figure 2J). In all, these data indicate that astroglial networks contribute to functional retinotopic maps specifically in the SC.

### Activity-dependent control of orientation representation and sensory-motor behavior by astroglial networks

**Figure 4:**
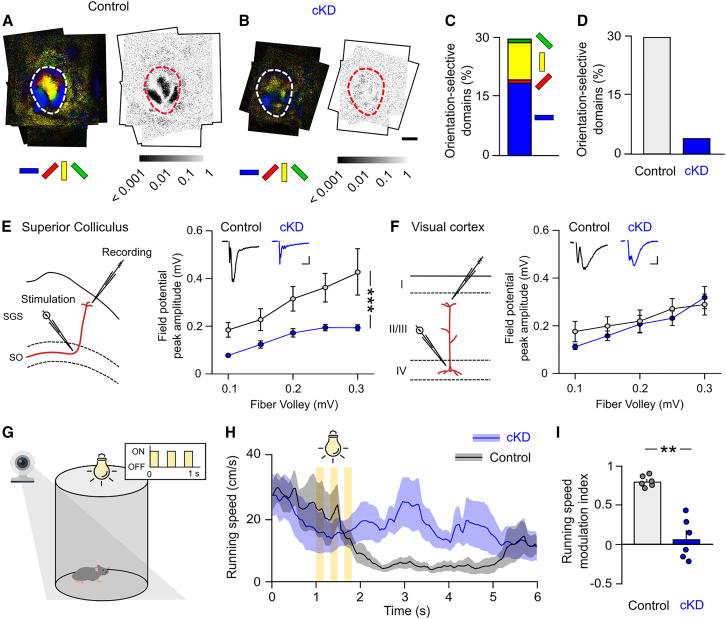
Activity-dependent control of orientation maps and light-induced temporary arrest behavior by astroglial networks (A and B) Averaged map of orientation in control (*n* = 5) (A) and cKD (*n* = 8) (B) mice. Reproducibility in functional organization across animals was tested with the Moore-Rayleigh test (right). Boundaries of the SC is depicted with the dotted line. Scale bar, 1 mm. (C) Representation of preferred orientations in control mice. (D) Quantification of collicular domains selective for orientation in control (gray) and cKD (blue) mice. (E and F) Left: schematics showing the location of the stimulation and recording pipette in the SC (E) and in V1 (F) to record field potentials. SGS, stratum griseum superficiale; SO, stratum opticum. Right: quantification of field potential peak amplitude in the SC (D) and in V1 (E) (two-way ANOVA, F(1,30) = 37.35, *p* < 0.0001) and (G) V1 (two-way ANOVA, F(1,32) = 1.085, *p* = 0.3053). (G) Schematic representation of the light-induced temporary arrest behavioral test. The mouse is placed in a closed arena. While the mouse is running, 3 flashes of white light (1 s total duration) is presented above the animal, causing the mouse to stop temporarily. (H) Averaged running speed following flashes presentation in control (*n* = 6) and cKD (*n* = 6) mice. (I) Quantification of the running speed modulation index. Normalized difference in running speed after light flashes relative to before light flashes (index = 1 indicates full stop) (control, *n* = 6; cKD, *n* = 6; *p* = 0.0022, *U* = 0, Mann-Whitney test). Data are shown as mean ± SEM.

We then investigated whether astroglial networks contribute in the SC to other functional maps, such as orientation preference (Figures 4A and 4B). This map has been reported in the SC of WT mice,^13^^,^^14^ but not in mouse V1, where neighboring neurons do not share the same selective features.^16^ In control mice, orientation maps were robust across animals and composed of patches responding almost exclusively to cardinal contours (Figures 4A and 4C). We found that disruption of astroglial connectivity in cKD mice impaired orientation maps (Figure 4B). Although we found some domains representing cardinal contours in the SC of cKD mice, these were not robustly represented across animals. The area of significant orientation-selective domains across animals was largely diminished in cKD mice compared to controls (Figure 4D). This indicates that SC astroglial networks also shape functional orientation maps. We found that the alteration of SC functional maps in cKD mice was associated at the circuit level with a specific impairment of the synaptic transmission of collicular neurons. Extracellular field recordings in the SC revealed a significant reduction (∼50%) in field potential amplitude in cKD compared to control mice (Figures 4E and 4F, F(1,30) = 37.35, *p* < 0.0001, two-way ANOVA), while no changes were observed in V1 (Figure 4F, F(1,32) = 1.085, *p* = 0.3053, two-way ANOVA). Given the large and selective impairment in functional visual maps and neuronal responses in the SC of cKD mice, we investigated whether this was associated with sensory-motor behavioral defects. For this purpose, we used an SC-dependent visually induced innate behavior, the light-induced arrest behavior assay (Figure 4G), which measures the temporary suspension of locomotion upon sudden flashes of light and has been proposed to relate to defense responses to aversive stimuli.^22^ In control mice, we found that the light flashes induced temporary arrest behavior characterized by a reduction of the running speed (Figure 4H) and a high-speed modulation index (0.8099 ± 0.06855, *n* = 6; Figure 4I). This temporary arrest behavior was markedly diminished in cKD mice (Figure 4H), with a significantly decreased speed modulation index (∼80%) compared to control mice (0.0763 ± 0.2591, *n* = 6, *p* = 0.0022, *U* = 0, Mann-Whitney test; Figure 4I). These results indicate that astroglial networks in the SC are key determinants of innate sensory-motor behaviors driven by visual stimuli.

## Discussion

Here, we show that astrocytes in the retinorecipient SC form unusually large gap junction-mediated networks, resulting from a high expression of gap junction proteins. Previous studies reported a relatively limited coupling in astrocytes from various brain areas, such as the barrel cortex (∼45 cells^23^), the somatosensory cortex (∼75 cells^24^), the hippocampus (∼130 cells^25^), or the olfactory bulb (<80 cells^26^), which is much lower than the size that we report here in the SC. Remarkably, we show that these astroglial networks are essential for driving the synaptic circuits underlying the functional visual maps in the SC, but not in V1. This has important functional consequences, as these astroglial networks contribute to sensory-motor responses, pointing to these networks as key regulators of visually induced behaviors.

Previous work on other sensory areas showed that astroglial networks are compartmentalized.^23^^,^^26^ This is apparent in the barrel cortex, in which astroglial networks stay within one barrel,^23^ as well as in the olfactory bulb, in which these networks are restricted to the glomeruli.^26^ The SC is a laminated structure in which the superficial layers are involved in visual processing.^27^ Surprisingly, we found that astroglial networks are not restricted to the visual layers, but instead also extend to deeper layers. This may result from the fact that the deeper SC receives inputs from auditory, somatosensory, but also visual areas and is thus involved in processing visual information.^28^ Interestingly, the retinotopic map observed in the visual layer of the SC is the reference spatial map for the other layers, such that SC maps of other sensory modalities are aligned to the retino-collicular map.^29^^,^^30^^,^^31^ Elucidating whether these uncompartmentalized astroglial networks contribute to this map alignment during development might uncover a coordinating function for astrocytes.

Most work on the influence of astrocytes on visual processing has been performed at the single-cell level. For instance, work on the ferret V1 revealed that astrocytes share the same orientation preferences as neighboring neurons.^4^ Additionally, astrocyte activation can alter orientation selectivity of adjacent neurons in mouse V1.^6^ Here, we reveal the importance of the network organization of astrocytes for information representation in visual maps in the SC. Remarkably, this astroglial regulation is specific to the SC, as it does not occur in V1. This region specificity could result from the heterogeneity of astroglial network size, which is much larger in the SC than in V1 due to the high expression levels of the gap junction channel proteins in the SC.

Functional maps are generally viewed as purely anatomical, based solely on neuron-to-neuron structural connections, but our study indicates that they are also influenced by other factors. We reveal that astrocytes are essential for functional maps and visual processing. We found that impairing the functional astrocyte network connectivity leads to deficits in retinocollicular transmission and visual processing, which indicates that the structural integrity of maps alone does not ensure their proper function. The anatomical presence of connections (maps in a structural sense) and their functional efficacy (maps in a functional sense) is thus crucial for information representation.

It is noteworthy that the SC is considered to coordinate complex behaviors involving the processing of sensory information for motor output. These behaviors include prey capturing^9^ and defense responses,^10^ which are crucial for survival. Given the large impairment in functional visual maps in the SC of cKD mice, this suggests that collicular astrocytic networks have implications in these complex behaviors. Consistent with this hypothesis, we found that disconnected astrocytic networks impaired an SC-dependent visually induced sensory-motor behavior, the light-induced arrest, thought to be related to defense behavior.^22^ In all, our work uncovers a role for the extensive uncompartmentalized astrocytic networks from the SC in driving the synaptic circuits underlying functional visual maps and behavior.

### Limitations of the study

We performed our study using mice of both sexes, thus precluding the identification of potential differential effects in male and female mice (extent of astroglial networks, functional visual maps, sensory-motor behavior). In addition, to investigate the role of astroglial networks in functional visual maps and sensory-motor behavior, we used astroglial conditional and inducible knockdown mice for both connexins (Cx30 and Cx43) (GFAP-creERT2 Cx30^fl/fl^/Cx43^fl/fl^, cKD). This approach does not permit us to evaluate the specific role of astroglial networks mediated by one connexin subtype (Cx30 or Cx43), known to have differential properties (expression, biophysical properties). Finally, we assessed the functional visual maps in anesthetized animals, which may underestimate the amplitude of the recorded signals.

## STAR★Methods

### Key resources table

**Table:** 

REAGENT or RESOURCE	SOURCE	IDENTIFIER
**Antibodies**		

Chicken-*anti*-GFP	Aves Labs	Cat# GFP-1020
Mouse-MBP	Novus Biologicals	Cat# NBP2-22121PE
Rabbit-Cx30	Life Technologies	Cat# 71-2200
Rabbit-Cx43	Invitrogen	Cat # 35-5000
Mouse-Cx43	BD Biosciences	Cat# 610062
Goat-*anti*-chicken 488	Invitrogen	Cat# A-11039
Goat-*anti*-mouse 555	Life Technologies	Cat# A28180
Donkey-*anti*-rabbit 647	Life Technologies	Cat# A-31573
Goat-*anti*-rabbit IgG	Cohesion Biosciences	Cat# CSA9004
Goat-*anti*-mouse IgG	Cohesion Biosciences	Cat# CSA9001
Primary anti-β-actin	Abcam	Cat# ab8227

**Chemicals, peptides, and recombinant proteins**		

Biocytin	Sigma-Aldrich	Cat# B4261;
Tamoxifen	Sigma-Aldrich	Cat# T5648
Paraformaldehyde (32%)	Electron Microscopy Sciences	Cat# 50-980-495
Triton X-100	Sigma-Aldrich	Cat# X100
Fluoromount-G	Southern Biotechnology	Cat# 0100-01
Alexa Fluor 555-conjugated streptavidin	Invitrogen	Cat# S21381
SDS	Euromedex	Cat# EU1-2026-50
Beta- glycerophosphate	Sigma-Aldrich	Cat#G9891
Orthovanadate	Sigma-Aldrich	Cat# S6508
Laemmli buffer	Euromedex	Cat#EU0006
PierceTM BCA kit	Thermofisher	Cat# 23227
Tris-buffered saline (TBS)	Euroedex	Cat# ET220
Tween 20	Sigma-Aldrich	Cat# P9416
Chemiluminescence detection kit- Western Lightning plus	Perkin Elmer	Cat# NEL103E001EA
Euthasol	Dechra	https://www.dechra.co.uk/products/livestock/prescription/euthasol
Urethane	Sigma-Aldrich	Cat# U2500
Chlorprothixene hydrochloride	Sigma-Aldrich	Cat# C1671
Ketamine (Imalgene 1000)	Boehringer Ingelheim	https://www.boehringer-ingelheim.com/fr/sante-animale/products/imalgene
Xylazine (Rompun 2%)	Elanco	https://www.myelanco.co.uk/products/rompun
Atropine	Sigma-Aldrich	Cat# Y0000878
Dexamethasone	MSD	https://www.msd-animal-health-hub.co.uk/Products/Dexadreson
NaCl solution (0.9%)	CDM Lavoisier	Cat# 201178
Tropicamide	Thea Lab	https://www.laboratoires-thea.com/en/monofreer-tropicamide
Neosynephrine,	Europhta	https://www.laboratoires-europhta.com/produit/neosynephrine-aa-faure
Lubrithal	Dechra	https://www.dechra.co.uk/products/cat/non-prescription/lubrithal

**Deposited data**		

Raw and analyzed data	This paper	Available upon request from lead contact

**Experimental models: organisms/strains**		

Mouse: C57BL/6J	Charles River	RRID: IMSR_JAX:000664
Mouse: GFAP-eGFP	F. Kirchhoff (University of Saarland, Germany)	Available upon request from lead contact
Mouse: Cx30 fl/fl Cx43 fl/fl	M. Cohen-Salmon (Collège de France, France) Pr. K. Willecke (University of Bonn, Germany)	Available upon request from lead contact
Mouse: GFAP-creERT2	F. Kirchhoff (University of Saarland, Germany)	N/A

**Software and algorithms**		

ImageJ (Version 1.54f)	Fiji	RRID: SCR_003070
Clampex 10	Molecular Devices	RRID: SCR_011323
Clampfit 10	Molecular Devices	RRID: SCR_011323
MATLAB (Version 2016a)	Mathworks	RRID: SCR_001622
CRS toolbox for ViSaGe	Cambridge Research Systems	https://www.crsltd.com/tools-for-vision-science/visual-stimulation/visage/
Python (Version 3.6)	Python Software Foundation	RRID: SCR_008394
DeepLabCut (Version 2.3.5)	The Mathis Lab	RRID: SCR_021398

### Resource availability

#### Lead contact

Further information and requests for resources and reagents should be directed to and will be fulfilled by the lead contact, Nathalie Rouach (nathalie.rouach@college-de-france.fr).

#### Materials availability

This study did not generate new unique reagents.

#### Data and code availability

• Original data generated in this study are available upon request from the lead contact, Nathalie Rouach (nathalie.rouach@college-de-france.fr).
• This paper does not report original code.
• Any additional information required to reanalyze the data reported in this paper is available from the lead contact, Nathalie Rouach (nathalie.rouach@college-de-france.fr).

### Experimental model and study participant details

#### Experimental design

The main objective of this study was to explore whether astroglial networks contribute to information representation by neuronal sensory maps and to sensory-motor behavior. To this end, we used the mouse SC, a midbrain structure from the visual system displaying functional maps and transforming sensory information into motor output. First, we investigated the spatial properties and connectivity of astroglial networks in the SC. Subsequently, we assessed the role of this astroglial networks connectivity in functional visual maps and its implication in SC-related visually induced innate behavior.

#### Animal studies

##### Mouse lines

All experiments were performed in accordance with the European Communities Council Directives of 01/01/2013 (2010/63/EU) for animal care and experimentation and of the French ethic committee (ethics approval #201902121059308 delivered by the French ministry of higher education, research and innovation). Experiments were carried out using mice of wildtype C57BL/6j background, mice expressing enhanced green fluorescent protein under the astroglial glial fibrillary acidic protein promoter (GFAP-eGFP), GFAP-creERT2 mice, as well as mice with conditional and inducible deletion of Cx30 and Cx43 in astrocytes that we generated (GFAP-creERT2 Cx30^fl/fl^Cx43 ^fl/fl^, cKD), as described below. C57BL/6j were obtained from Janvier labs (France), GFAP-eGFP and GFAP-creERT2 mice were provided by F. Kirchhoff (University of Saarland, Germany), Cx43 ^fl/fl^ mice by K. Willecke (University of Bonn, Germany) and Cx30 ^fl/fl^ mice by M. Cohen-Salmon (College de France, France), and were all characterized.^32^^,^^33^^,^^34^^,^^35^ Mice were backcrossed to the C57BL/6J background and were housed under standard conditions (12-h light/12-h dark cycle, light on at 7 a.m., 22 ± 1°C ambient temperature, 60% relative humidity), with *ad libitum* access to food and water. Adult mice of both genders were used at postnatal days 50–100. All efforts were made to minimize the number of animals used and their suffering.

###### Generation of Cx30 and Cx43 conditional knockdown mice

Astroglial Cx30 and Cx43 conditional and inducible mice were generated by crossing the GFAP-creERT2 line expressing the cre-recombinase transgene driven by the astrocytic glial fibrillary acidic protein (GFAP) promoter^35^ with Cx30 ^fl/fl33^ and Cx43 ^fl/fl34^ lines containing cre-excisable *loxP* sequences in the endogenous *Gjb6*^33^ and *Gja1*^34^ genes, respectively. Tamoxifen (10 mg/mL in corn oil, Sigma) was injected intra-peritoneally in GFAP-creERT2 (Control) and GFAP-creERT2 Cx30^fl/fl^Cx43 ^fl/fl^ (cKD) mice (100 mg/kg body weight) during four consecutive days, and experiments were performed at least three weeks after the last injection.

### Method details

#### Slice electrophysiology

After rapid extraction of mouse brains, coronal slices (300-400μm) containing the SC were cut using a vibratome (Leica VT1200S) in ice-cold artificial cerebrospinal fluid (aCSF) composed of (in mM): 119 NaCl, 2.5 KCl, 2.5 CaCl_2_, 1.3 MgSO_4_, 1 NaH_2_PO_4_, 26.2 NaHCO_3_ and 11 glucose. Slices were allowed to recover for a minimum of 30min in a chamber containing aCSF at room temperature. ACSF was continuously bubbled with 95% O_2_/5%CO_2_. Slices were placed into a submersion-type recording chamber that was mounted on an Olympus BX51WI microscope. Slices were continuously perfused with aCSF at room temperature (2 mL/min). To examine electrophysiological properties of astrocytes and the size of the astroglial network, patch-clamp experiments were performed in the superficial visual layers of the SC and in V1. Whole-cell patch-clamp electrophysiological recordings were performed using a glass pipette (3-5MΩ) filled with intracellular solution composed of (in mM): 105 potassium gluconate, 30 KCl, 10 HEPES, 0.3 EGTA, 4 ATP-Mg, 0.3 GTP-Tris, and 10 phosphocreatine (pH adjusted to 7.4 with KOH, 280 mOsm). During experiments, a depolarizing ramp protocol was performed in voltage-clamp (from −200 to 40mV) to assess astroglial electrophysiological passive currents. Astrocytes were identified by their small soma, low membrane resistance, passive currents and absence of action potentials.^17^^,^^23^ During the recording, cells were discarded when the membrane potential or resistance varied more than 10%. For dye coupling experiments, the intracellular solution contained biocytin (7 mg/mL, Sigma), a gap junction permeable tracer, which diffused in the network during 20 min. For extracellular field potentials recordings in the SC, a stimulation electrode containing aCSF solution was placed in the *stratum opticum* (optical layer) while the recording electrode containing a 1M NaCl solution was placed in layer *stratum griseum superficiale* (superficial gray layer), while for V1, the stimulation electrode was placed in layer IV and the recording electrode was placed in layer II/III. Stimulus artifacts were blanked in sample traces. Analysis was performed by comparing the amplitude of the presynaptic fiber volley (input) to the peak amplitude of the field potential. Recordings were acquired with MultiClamp 700B amplifier (Molecular Devices), digitized at 10 kHz, filtered at 2 kHz, stored and analyzed on computer using Clampex 10.2 and Clampfit softwares 10.3 (Molecular Devices).

#### Immunohistochemistry and confocal imaging

##### Immunohistochemistry

For immunohistochemical assessment of Cx30 and Cx43, GFAP-eGFP mice were anesthetized with a lethal dose of Euthasol (150 mg/kg, intraperitoneal injection (i.p.)), and transcardially perfused with phosphate buffered saline (PBS) followed by 2% paraformaldehyde (PFA) in PBS. After perfusion, brains were carefully dissected and post-fixed for 24 h in 2% PFA followed by 24 h in 30% sucrose solution for cryoprotection. Brain coronal sections (40μm) of slices containing both the SC and V1 were cut with a freezing microtome (Epredia HM 450, ThermoScientific) and stored in PBS. Free floating slices of the SC and V1 were incubated for 2 h with PBS, 1% gelatine and 0.25% Triton X-100 (PGT 0.25%) to block unspecific binding sites. Brain sections were incubated at 4°C with the following primary antibodies: anti-GFP (Chicken, 1:500, Aves Labs), myelin basic protein (MBP) (mouse, 1:300, Novus Biologicals), and Cx30 (rabbit, 1:500, Life Technologies) or Cx43 (rabbit, 1:500, Invitrogen). 24 h later, the slices were washed three times in PGT 0.25% followed by an incubation of 2 h at room temperature with the following secondary antibodies: goat-*anti*-chicken 488 (1:1000, Invitrogen), goat-*anti*-mouse 555 (1:1000, Life Technologies) and donkey-*anti*-rabbit 647 (1:1000, Life Technologies). Finally, the slices were washed several times in PBS and mounted with Fluoromount-G (Southern Biotechnology). To visualize the extent of astroglial networks, slices were then fixed with 4% PFA overnight, and subsequently incubated in PBS, 1% gelatine and 1% Triton X-100 (PGT 1%) followed by revelation using Alexa Fluor 555-conjugated streptavidin (1:300 in PGT1%, Invitrogen). After several PBS washes, slices were mounted in Fluoromount-G (Southern Biotechnology).

Slices were examined with a confocal laser-scanning microscope (Leica DMI6000 Inverted SP5). For dye coupling experiments, cells were examined with a 20×/0.75NA objective. Z-stacks of consecutive confocal images taken at 0.5μm increments were acquired with a Diode-pumped solid-state laser (DPSS) 561 nm laser controlled by LAS AF software (Leica). Cell counting was performed using ImageJ software that also provided x and y coordinates of the cells within an image. This information was used to determine the coupling spread in the visual layer of the SC into the medio-lateral and dorsoventral directions using MATLAB. For immunostaining, the area of interest was first identified based on the MBP staining with a 10x/0.3 objective using the 561nm DPSS laser. Subsequently, two to three images were taken using a 63x/1.40 objective with a 488nm argon, 561nm DPSS and 633nm Helium-Neon (HeNe) lasers. Z-stacks of 10μm were acquired with 0.5μm increments starting 5μm under the surface. Images were analyzed using ImageJ Software. Lastly, the integrative density was used to assess Cx30 and Cx43 fluorescence.

#### Immunoblotting

To study region-specific Cx30 and Cx43 expression patterns, SC and V1 samples were harvested from 500μm sections sliced using a microtome (Leica VT1200S) and quickly frozen on dry ice. Samples were immersed in 2% Sodium Dodecyl Sulfate (SDS) containing protease inhibitor cocktail (Euromedex), phosphatase inhibitors (Beta-glycerophosphate, 10 mM) and orthovanadate (1mM). After lysing by sonication (Ultrasonic cell disrupter, Microson), the samples were centrifuged (13 000 rpm at 4°C) for 10 min. The supernatant was collected, 5x Laemmli buffer was added and boiled for 5 min. Protein concentration was determined using the PierceTM BCA kit (Thermofisher scientific). For each sample, 20μg of protein was separated on Bis-Tris 4-12% NuPAGE gels and transferred onto nitrocellulose membranes. Non-specific binding sites were blocked by incubating the membranes in Tris-buffered saline (TBS)-Tween-milk solution (500 mL TBS 1X, 500μL Tween 20X, 25g non-fat powder milk) for 1h. Then, the membranes were incubated overnight at 4°C with primary antibodies polyclonal Cx30 (rabbit, 1:500, Life Technologies) and monoclonal Cx43 (mouse, 1:500, BD Biosciences). After appropriate washing, they were incubated with HPR-conjugated secondary antibodies: goat-*anti*-rabbit IgG (1:2500, Cohesion Biosciences) and goat-*anti*-mouse IgG (1:2500, Cohesion Biosciences). The HRP-conjugated primary anti-β-actin antibody (1:2000, ABCAM) was used as a loading control. Immunosignals were revealed with the chemiluminescence detection kit (Western Lightning plus-ECL, NEL103E001EA, PerkinElmer). Semi-quantitative densitometric analysis was performed with the ImageJ software. The same procedure was used to determine Cx30 and Cx43 protein levels in control and cKD mice in the SC and visual cortex.

#### Intrinsic optical imaging

##### Surgery

Control and cKD animals were anesthetized using a combination of an anesthetic (urethane, 1.2 g/kg, i.p.) and a sedative (chlorprothixene, 8 mg/kg, intramuscular (i.m.)). Atropine (0.1 mg/kg) and dexamethasone (2 mg/kg) were injected subcutaneously. After sufficient depth of anesthesia, animals were head-fixed in a stereotactic frame. Temperature was maintained at 37°C by rectal temperature monitoring. During surgery, eyes were moisturized by 0.9% NaCl solution. In-between recordings, eyes were regularly checked for opacity. To reach the SC, we performed a craniotomy and gently aspirated the cortex above.^12^^,^^14^ The visual cortex, on the other hand, was recorded through the intact skull after exposing the cranium contralateral to the stimulated eye. For both the SC and visual cortex, the area was covered by 2.5% agarose and a glass coverslip.

##### Set up

A Dalsa 1M60 CCD camera was tilted parallel to the collicular or cortical surface and the focal plane was set 250μm below the surface for the SC and 400μm for the VC. Images were acquired after a 2 × 2 pixels spatial binning with a resolution of around 10.5μm/pixel using a 135 × 50 mm tandem lens (Nikon) configuration. Intrinsic signals were acquired with a 700nm illumination wavelength.

##### Visual stimulation

Visual stimuli were displayed on a 21″ LCD monitor located 20 cm in front of the eye contralateral to the hemisphere being imaged so that the screen was covering 110° of azimuth and 60° of altitude. Coordinates of the monitor were reprogrammed in order to maintain spatial and temporal frequencies constant across the visual field’s eccentricities. To record retinotopic maps, we used continuous, periodic imaging methods.^36^ For the azimuth map, a vertical bar drifting rightwards along the horizontal axis of the screen for 8 s was presented 30 times to the animal (4 min in total). During the second session, the bar was drifted leftwards. For the elevation map, the same protocol was applied with a horizontal bar drifting downwards and upwards along the vertical axis of the screen.

To record the orientation map, also a continuous, periodic stimulation protocol was used.^36^^,^^37^ A drifting, orientated sine-wave was rotated with an angular speed of the rotation was 2 rotations per minute (rpm). The drift of the gratings was set at 1.5 Hz and the spatial frequency at 0.015 cpd. The stimulus was first rotated counter clockwise for 20 cycles (10 min) and then clockwise for another 20 cycles.

#### Optical imaging analysis

##### Retinotopic maps

To reconstruct retinotopic maps, slow varying components independent of the stimulation were first subtracted by applying the generalized indicator function method for each session.^38^ Fourier transform was then performed on the temporal signal at each pixel to extract phase and magnitude related to the frequency of stimulation 1/8 Hz. The azimuth retinotopic map was calculated as half of the difference between the phase maps obtained for the bar drifting rightward and leftward.^36^ Similarly, the elevation map was calculated as half of the difference between the phase maps obtained for the bar drifting downward and upward.

##### Orientation map

To reconstruct the orientation map, the generalized indicator function method was first applied for each recording session.^38^ Then, Fourier transform was performed on the temporal signal at each pixel to extract phase and magnitude at half the frequency of rotation (f_rot_/2 = 1/15 Hz) to obtain phase maps of the anticlockwise (Φ+) and clockwise (Φ-) rotations. This average value was subtracted from Φ+ and Φ- for each animal to account for the hemodynamic delay.

##### Registration of functional maps among animals

To ensure precise alignment of functional maps among animals, the maps of retinotopy were rotated and shifted to minimize least square error in azimuth and elevation representation across animals. Maps of retinotopy and orientation were then averaged in these new coordinates and the Moore-Rayleigh test was performed at each pixel to determine the reproducibility of functional maps across animals. The averaged maps of elevation and azimuth were used to define the border of the SC or visual cortex.

#### Light-induced temporary arrest behavioral assay

To assess light-triggered behaviors in freely moving animals,^22^ mice were placed into a closed cylindrical arena of 30 cm diameter and 50 cm height installed in a chamber designed to isolate mice from environmental noise. A white LED source was positioned above the arena and a camera (Sony Effio-E 700TVL) was used to record the location of the animal. Each mouse was allowed a habituation period in the arena for 3 min prior to the behavioral test in a 30 Lux environment. During the test, while the mouse was in motion, the LED source emitted three flashes of white light (100 Lux on the ground) lasting a total of 1 s, causing the mouse to pause momentarily. Video recordings were conducted at a frame rate of 25 frames per second. Analysis was performed with Python using DeepLabCut Version 2.3.5,^39^ which facilitates the estimation of specific mouse body parts within video recordings. The software’s pose estimation capabilities were harnessed using a convolutional neural network architecture based on ResNet-50.^40^^,^^41^ The training process involved 500,000 iterations with a batch size of one, adhering to the default hyperparameters provided by DeepLabCut. The network was then trained with manually annotated frames that delineated key anatomical landmarks of the mouse: the snout, the barycenter and the base of the tail. To do this, a total of 420 frames were extracted from the 12 video sequences using a k-means clustering approach to ensure a representative training set. These frames were annotated by a single experienced observer to maintain consistency. To augment the training data and enhance the model’s generalizability, we employed the imgaug library.^42^ 95% of labeled frames were then used for training and the remaining 5% for validation of the model. For the analysis of movement, we computed the velocity of each tracked body part using a Savitzky-Golay filter.^43^ Body parts with a likelihood inferior to 0.9 were removed. A subsequent smoothing over a window of five frames was applied to mitigate short-term fluctuations. Data representing the barycenter of the mouse was used for further quantification. For this purpose, the speed profile of each animal was aligned to the onset of the first flash of light. The baseline running speed V_baseline_ was calculated as the averaged running speed within a 1 s window immediately before the first flash of light. The running speed induced by the stimulation V_arrest_ was calculated as the averaged running speed within a 3 s window 1 s after the first flash of light. The running speed modulation index was defined as (V_baseline_-V_arrest_)/V_baseline._

##### Electroretinography

Electroretinograms (ERG) were performed in mice. For dark adaptation, mice were kept overnight in the darkness. The next morning, the animals were anesthetized with ketamine/xylazine (i.p., 80 mg/kg; 8 mg/kg, Axience). Tropicamide (Thea Lab) and phenylephrine (Neosynephrine, Europhta) were applied for pupil dilation. Temperature was maintained at 37°C by a heating pad. To keep the eyes open during the recording, upper and lower eye lids were retracted. A gold-loop electrode was placed on the corneas and maintained with lubrithal (Dechra) to record ERG (SIEM Bio-medicale). Reference and ground electrodes were respectively placed on the head and tail. The light stimulus was provided in a Ganzfeld with increasing flash intensity (0.04, 0.32, 3.19, 8 cd s/m2) for scotopic conditions and a single intensity (8 cd s/m2) for photopic stimulation. Each scotopic ERG response represents the average of five responses from a set of five flashes of stimulation. Each cone photopic ERG response represents the average of ten responses to a set of ten consecutive flashes. Amplitudes of scotopic a- and b-waves and photopic b-waves were measured at the maximum negative or positive peaks of the recording with respect to the baseline before stimulation.

### Quantification and statistical analysis

All data are expressed as mean ± SEM unless otherwise stated and n represents the number of independent replicates. For all datasets, outliers were identified using the ROUT method (Q = 1%).^44^ For statistical comparison, normality test as well as variance analysis were performed, and the appropriate two-sided statistical parametric or non-parametric test was used. Two-tailed unpaired or paired tests were used for between-group comparisons. Statistical significance for within-group comparisons was determined by one-way or two-way ANOVAs followed by post hoc tests. For the intrinsic optical imaging data, the Moore-Rayleigh test was performed to determine the reproducibility of functional maps across animals using MATLAB. Appropriate sample sizes were based on best practices in the literature as well as on ethical standards to minimize numbers of animals for experiments, and were dictated by the magnitude of experiment-to experiment variation. All statistical analysis was performed in GraphPad Prism and MATLAB. P-values were considered significant when *p* < 0.05 (^∗∗∗^*p* < 0.001, ^∗∗^*p* < 0.01, ^∗^*p* < 0.05).

## Supplemental information

Document S1. Figures S1–S6

Document S2. Article plus supplemental information
