# Supplementary material for: Astroglial networks control visual responses of superior collicular neurons and sensory-motor behavior
Source: Cell Rep. 2024 Jul 13;43(7):114504. doi: 10.1016/j.celrep.2024.114504 (PMC11290320; doi:10.1016/j.celrep.2024.114504)
Supplement: Document S1. Figures S1–S6 [file mmc1.pdf]

Cell Reports, Volume *43*

Supplemental information

Astroglial networks control visual responses of superior collicular neurons and sensory-motor behavior

Josien Visser, Giampaolo Milior, Rachel Breton, Julien Moulard, Maina Garnero, Pascal Ezan, Jérôme Ribot, and Nathalie Rouach

Supplemental information

This PDF file includes:

Figs. S1 to S6

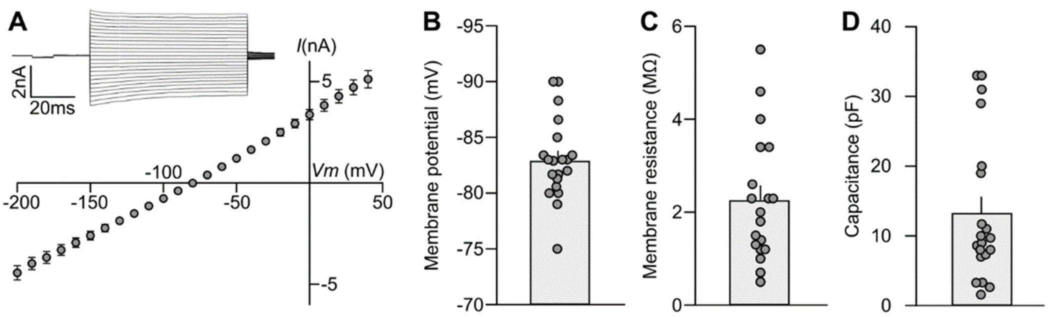

Fig. S1. Electrophysiological properties of astrocytes in the visual layers of the SC. Related to Figure 1. (A) Average current-voltage relationship (I-V curve) (n=16) shows that astrocytes in the visual layer of the SC display characteristic passive currents. A representative astrocyte I-V curve is shown (top). **(B)** Quantification of intrinsic electrophysiological properties of astrocytes in the visual layers of the SC (membrane potential, membrane resistance and capacitance, n=19-20).

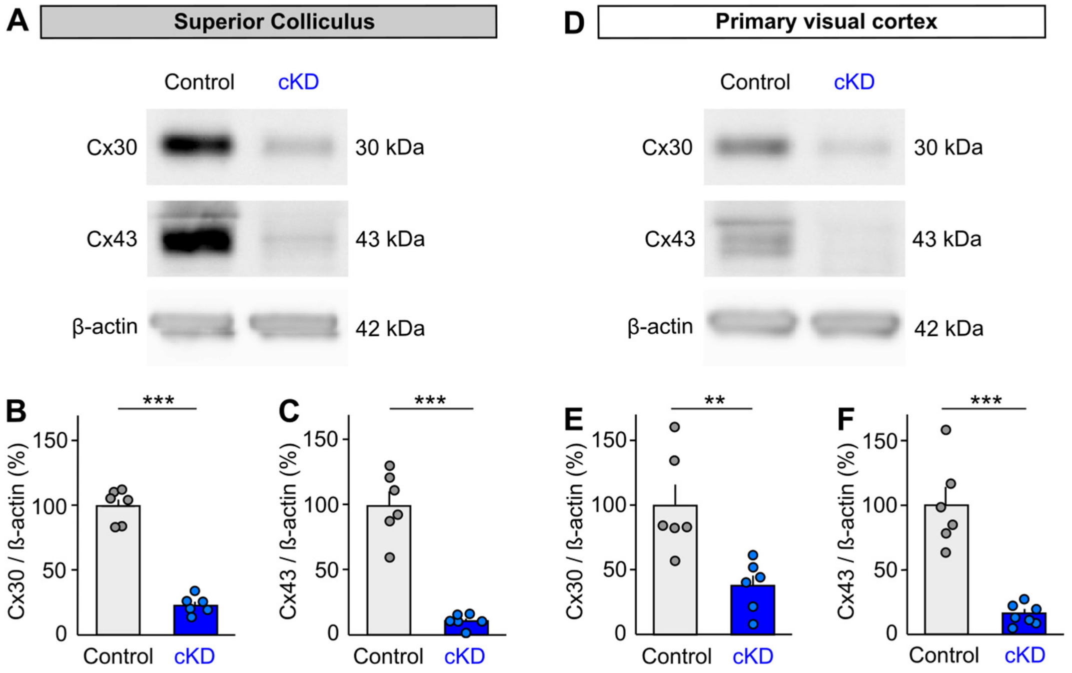

Fig. S2. Strong reduction of astroglial connexins expression levels in cKD mice. Related to Figure 2. (A) Representative western blots for Cx30, Cx43 and β-actin protein levels in the SC of control and cKD mice. **(B-C)** Quantification shows massive decrease in Cx30 **(B)** and Cx43 **(C)** levels in the SC of cKD mice (n=6) compared to control (n=6). Connexin protein levels are normalized to β-actin levels and to the expression of control mice (Cx30, $t(10) = 12.61$, ***$p<0.0001$; Cx43, $t(10) = 8.335$, ***$p<0.0002$, unpaired t-test). **(D-F)** Same as **(A-C)** for the primary visual cortex (Cx30, $t(10) = 3.516$, **$p=0.0056$); (Cx43, $t(10) = 5.891$, ***$p=0.0002$, unpaired t-test).

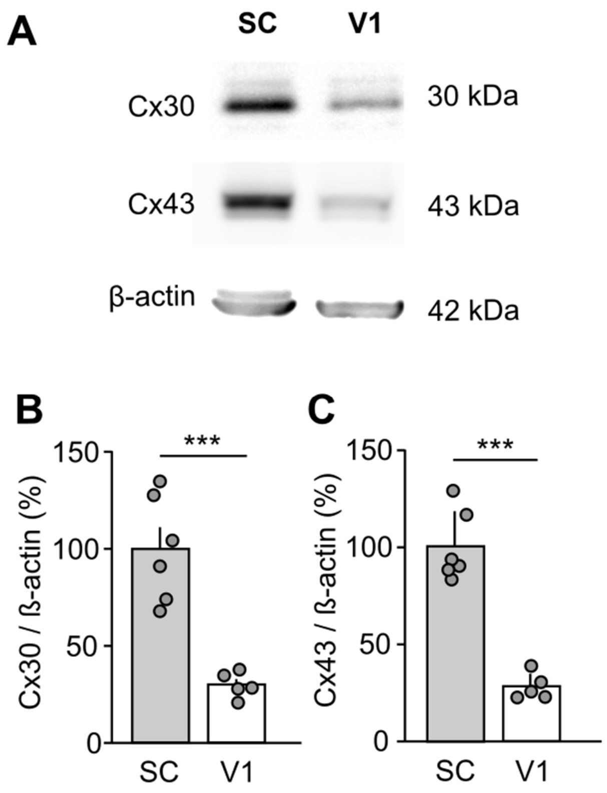

Fig. S3. Strong expression of Cx30 and Cx43 in the SC analyzed by western blot. Related to Figure 2. (A) Representative western blot for Cx30, Cx43 and β-actin protein levels in the SC and V1. **(B-C)** Quantification of Cx30 **(B)** and Cx43 **(C)** expression in the SC (n=6) compared to V1 (n=5). Expression of Cxs were normalized to β-actin and normalized to the SC (Cx30, $t(9)=5.525$, ***$p=0.0004$); (Cx43, $t(9)=8.306$, ***$p<0.0001$, unpaired t-test).

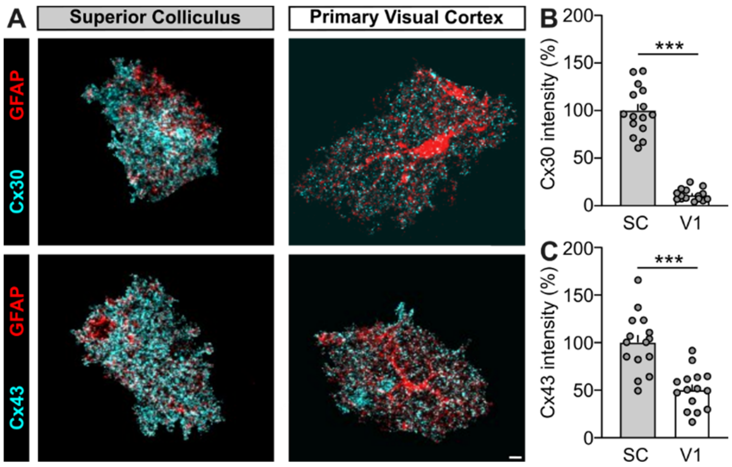

Fig. S4. Strong expression of Cx30 and Cx43 per astrocyte in the SC. Related to Figure 2. (A) Representative images of single astrocytes visualized by GFAP staining (red) and Cx30 and Cx43 staining (cyan) in the SC (left) and in V1 (right). Scale bar, 15 µm. **(B**, **C)** Quantification of Cx30 (**B**) and Cx43 (**C**) levels per astrocyte in the visual layers of the SC (n=15) and in V1 (n=15). (Cx30, $t(28)=12.54$, ***$p<0.0001$; Cx43, $t(28)=5.142$, ***$p<0.0001$, unpaired t-test).

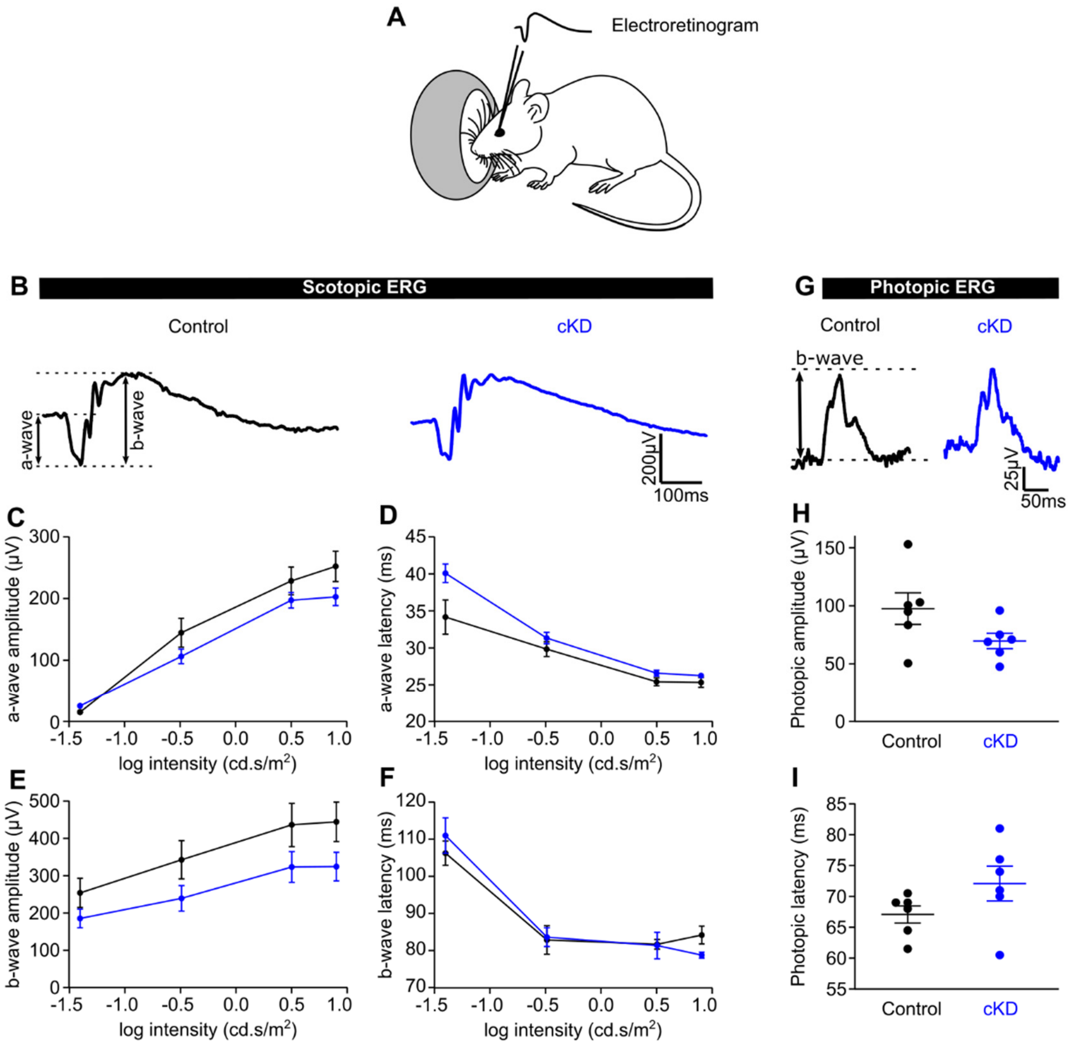

Fig. S5. Normal electroretinogram responses in mice with disconnected astrocytes. Related to Figure 3. (A) Schematic representation of electroretinogram (ERG) recordings in mice. ERGs were performed under **(B-F)** scotopic and **(G-I)** photopic conditions. **(B)** Representative retinal potential responses upon the highest intensity light flash (8cd.s/m^2) in control (black) and cKD mice (blue). **(C-F)** Quantification of scotopic ERG responses. No difference was observed between control (n=6) and cKD (n=6) mice in **(C)** the a-wave amplitude (2-way ANOVA, F(3,40)= 1.242, p=0.3074), **(D)** the a-wave latency (2-way ANOVA, F(3,40) = 2.348, p=0.0870), **(E)** the b-wave amplitude (2-way ANOVA, F(3,40)= 0.1346), p=0.9388 and **(F)** the b-wave latency (2-way ANOVA, F(3,40)= 0.9234, p=0.4383). **(G)** Representative photopic ERG response after a 8 cd.s/m^2 flash light stimulus recorded from control (black) and cKD (blue) mice. No difference was observed between control (n=6) and cKD (n=6) mice in **(H)** the b-wave amplitude (t-test, t(10) = 1.1843, p=0.0951) and **(I)** the b-wave latency (t-test, t(10) = 1.592, p=0.1425) (n=6 animals per group).

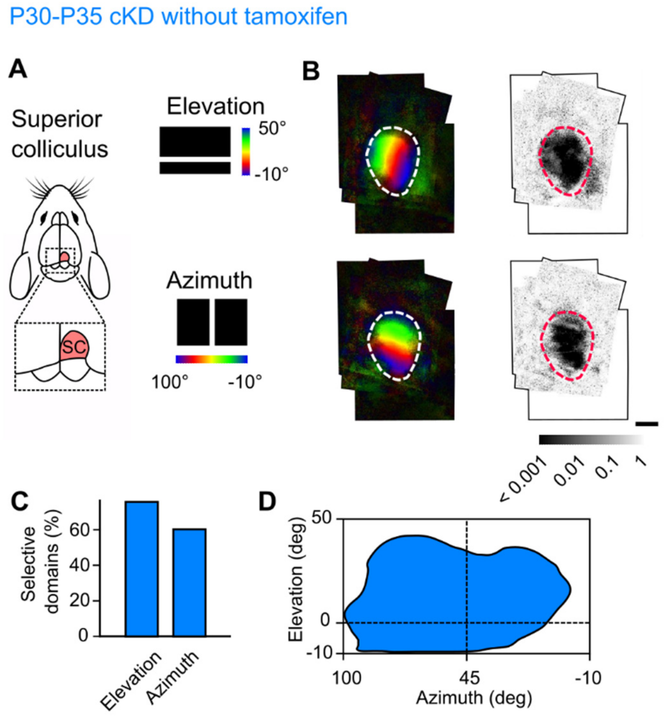

Fig. S6. Normal retinotopic maps in the SC of cKD mice prior tamoxifen injection. Related to Figure 3. (A) Schematic representation of the SC imaged with intrinsic optical imaging**. (B)** Averaged retinotopic maps of elevation (top panels) and azimuth (bottom panels) in the SC for cKD (n=5) mice at P30-P35 prior tamoxifen injection. Reproducibility in functional organization across animals was tested with the Moore-Rayleigh test (right panels). Boundaries of the SC is depicted with dotted line. Scale bar, 1 mm. **(C)** Quantification of collicular domains selective for elevation and azimuth. **(D)** Representation of visual locations selective for both elevation and azimuth.
